# Magnetic Resonance Image-Guided Biopsies with a High Detection Rate of Prostate Cancer

**DOI:** 10.1100/2012/975971

**Published:** 2012-03-12

**Authors:** Dirk G. Engehausen, Karl Engelhard, Siegfried A. Schwab, Michael Uder, Sven Wach, Bernd Wullich, F. Steffen Krause

**Affiliations:** ^1^Department of Urology, University Hospital Erlangen, Friedrich-Alexander University of Erlangen-Nuremberg, Krankenhausstrasse 12, 91054 Erlangen, Germany; ^2^Institute of Radiology, Martha-Maria Hospital, Stadenstrasse 60, 90491 Nuremberg, Germany; ^3^Institute of Radiology, University Hospital Erlangen, Friedrich-Alexander University of Erlangen-Nuremberg, Krankenhausstrasse 12, 91054 Erlangen, Germany

## Abstract

*Aim*. To explore the potential of transrectal magnetic resonance image- (MRI-) guided biopsies of the prostate in a patient cohort with prior negative ultrasound guided biopsies. *Patients and Methods*. Ninety-six men with suspected prostate cancer underwent MRI-guided prostate biopsies under real-time imaging control in supine position. *Results*. Adenocarcinoma of the prostate was detected in 39 of 96 patients. For individual core biopsies, MRI yielded a sensitivity of 93.0% and a specificity of 94.4%. When stratifying patients according to the free-to-total prostate-specific antigen (PSA) ratio, the prostate cancer discovery rate was significantly higher in the group with ratios less than 0.15 (57.1%). *Conclusion*. MRI-guided biopsy of the prostate is a diagnostic option for patients with suspected prostate cancer and a history of repeatedly negative transrectal ultrasound-guided biopsies. Combined with the free-to-total PSA ratio, it is a highly effective method for detecting prostate cancer.

## 1. Introduction

Digital rectal examination, transrectal ultrasound (TRUS), serum PSA assay, and TRUS-guided prostate biopsy are common methods used for prostate cancer evaluation. In addition, the feasibility of MR imaging, MRSI, and DCE-MRI in the diagnostic process of prostate cancer have been reported in several studies [1, 2]. MRI is capable of displaying the prostate in the T2-weighted image with its zonal anatomy and tumor-suspected areas within the high signal peripheral zone. The reported sensitivity for the detection of prostate cancer by MRI varies between 57% and 100% and the specificity between 44% and 96% for predicting positive biopsies [3]. Although the results differ somewhat, endorectal MRI seems to offer notably higher sensitivity compared to TRUS for the detection of prostate cancer. Consequently, there is increasing interest in using this MRI method for prostate cancer diagnosis in patients with suspected prostate cancer and prior tumor-negative TRUS-guided prostate biopsies. In 2000, endorectal MRI-guided biopsy of the prostate was first described as a transperineal puncture in one man [4]. In the meantime, other studies have been published, yet most of them report on only small numbers of patients [5, 6]. One early study comprised 12 patients, who had received MRI-guided prostate biopsy in a prone position [7]. In 2010, Hambrock reported results from the largest series, until now, with 68 patients using a 3 Tesla Trio Tim MR scanner [8]. In the present study, we report our results from endorectal MRI-guided prostate biopsies in 96 patients, which were performed in a supine position.

## 2. Patients and Methods

### 2.1. Study Population and Histological Evaluation

After approval from the local ethical committee, a total of 96 men underwent endorectal MRI-guided prostate biopsy between December, 2003 and December, 2007. Informed consent was retrieved from every participant. All of these patients had previously undergone TRUS-guided prostate biopsies (1 to 6 sessions) with tumor-negative results and a continuing clinical suspicion of prostate cancer. Patients were excluded if they had any of the usual contraindications to MRI (e.g., cardiac pacemakers or other metallic implants). No further patient selection was applied. Histological examination of the core biopsies was performed by experienced pathologists using hematoxylin-eosin staining on formalin-fixed, paraffin-embedded sections. Immunohistochemical studies, using the basal cell marker p63 and P504S/alpha-methylacyl-coenzyme A racemase, were performed in cases that were not clearly cancerous based on morphology alone.

### 2.2. MRI and Prostate Biopsy Technique

All patients underwent diagnostic MRI with an endorectal body phased-array coil before undergoing MRI-guided biopsy in a 1.0 Tesla (T) (*n* = 31) or 1.5 T (*n* = 66) MRI scanner under real-time MR-imaging control. The criteria used for malignancy in MRI were reported recently by Engelhard et al. [5]. We analyzed T2-weighted images to identify profoundly suspect or moderately suspect target regions. However, T2-weighted imaging has significant limitations for depicting cancer, especially in the transitional and central zone as well as in the anterior part of the peripheral zone, because cancer and normal tissues both have low signal intensity on T2-weighted images in these areas. In addition, low signal intensity may be seen in the peripheral zone on T2-weighted images in the presence of many noncancerous abnormal conditions, such as nonspecific inflammation, biopsy-related hemorrhage, postradiation fibrosis, and changes following hormone deprivation therapy [9, 10]. Therefore, we defined profoundly tumor-suspect regions within the normally high signal peripheral zone as asymmetric hypointense lesions. In the front gland (transitional zone and central zone) moderately tumor-suspect regions were defined as low signal intensity lesions depending on the circumjacent tissue when they were enclosed by structures with higher signal within the scope of an adenomatous hyperplasia [5].

**Figure 1:**
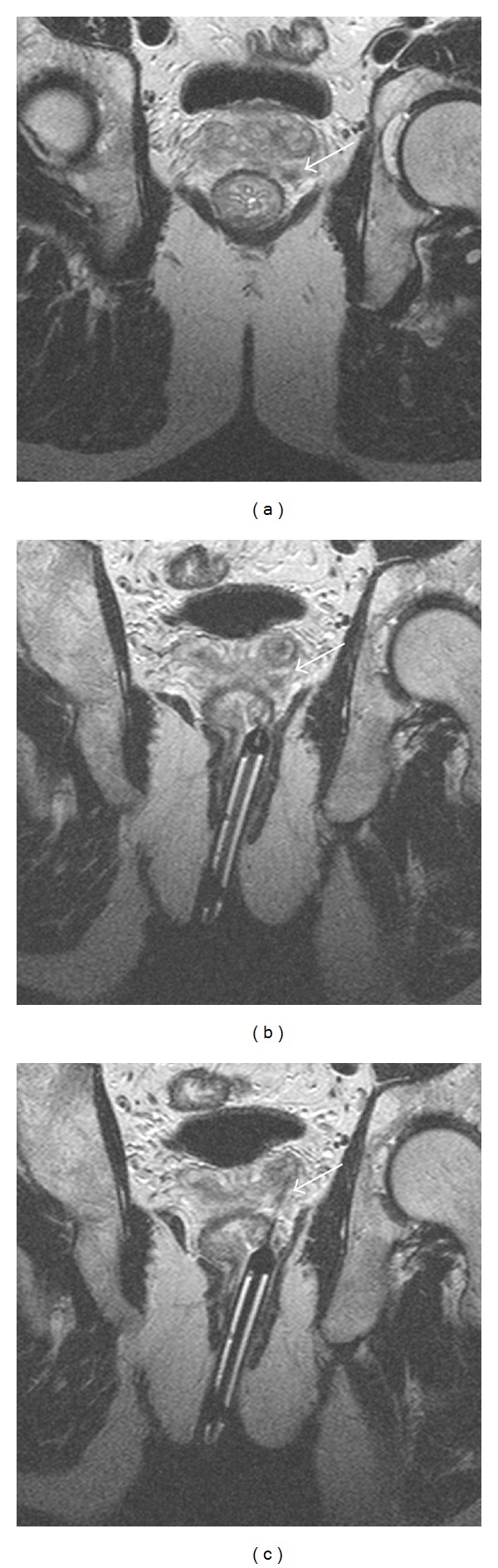
Prostate biopsy in a 1.5-T MRI system. (a) MR-image of a prostate gland with tumor-suspected area. (b) Prostate gland with needle guidance being positioned towards the tumor-suspected area. (c) Biopsy needle in the tumor-suspected area. In every image, the tumor-suspected area is marked by an arrow.

Prostate biopsy was performed with the patients positioned in a supine position within a closed 1.0- or 1.5-T MRI system (MAGNETOM Symphony, Siemens Medical Solutions, Erlangen, Germany) as already described [5]. A prophylactic antibiotic therapy was given (Ciprofloxacin 250 mg 1-0-1/d) for 5 days, starting 2 days before the procedure. No patient received general anesthesia or sedation, only mucosal infiltration analgesia of the rectum was administered (Mepivacainhydrochlorid 1%, 2 mL). After localization of the tumor-suspected area, the MR-visible biopsy needle guide was inserted into the patient's rectum and guided to the area to be punctured, as shown in Figure 1. The biopsies were performed using an MR-compatible biopsy gun (fully automatic BiopsyGun—16 G, INVIVO Germany, Schwerin, Germany). The correct position of the biopsy needle in the target area was documented. Two-to-six biopsy cores from each patient were extracted with at least one from the suspected area and at least one negative control biopsy. These negative control biopsies were extracted from areas without tumor-suspicious lesions, preferably contralateral to the major suspicious area. The MRI intervention took 40 to 60 minutes per patient.

### 2.3. Statistical Analysis

The median and range were used to describe the data, and nonparametric test statistics were applied. Prevalence, sensitivity, and specificity were calculated by 4 field charts. Fisher's exact test was used for comparison of prostate cancer detection rates between the group with free-to-total PSA ratios <0.15 and the group with ratios ≥0.15. Population characteristics between patients with or without prostate cancer were compared using Mann-Whitney *U*-test statistics. The ability of the free-to-total PSA ratio to discriminate between patients with or without prostate cancer was analyzed using receiver operating characteristics. The cutoff value with the best overall performance was selected according to the respective likelihood ratio. Statistical significance was considered at *P* values ≤0.05. Statistical analyses were performed using Prism 4.0 software (GraphPad Inc, La Jolla, Calif, USA). A core biopsy taken from an area suspected for malignancy by MRI was considered to be true-positive when the imaging results were confirmed by histology. Likewise, core biopsies taken from areas not suspected by MRI were considered to be true negative when histology did not show malignancy.

## 3. Results

A total of 96 men underwent MRI-guided prostate biopsy in the supine position.  Table 1 lists the patient demographic and tumor characteristics. There was information on the PSA isoforms for 69 patients, with free PSA ranging from 0.12 to 5.64 ng/mL (median 1.24 ng/mL) and the free-to-total PSA ratio ranging from 0.04 to 0.5 (median 0.14).

### 3.1. Analysis of MRI-Guided Core Biopsies

During MRI intervention, an average of 4.3 biopsy cores (range 2 to 6) were extracted per patient (Figure 1). Prostate cancer was histologically confirmed in 39 of the 96 patients, corresponding to a detection rate of 40.6%. Patients with tumor-negative histology results were transferred to a continuous surveillance scheme with a median follow-up time of 1.7 years (range: 2 months–4.5 years). In ten of these 57 patients, prostate cancer was detected within an interval of three years after MRI intervention. Of these, prostate cancer was identified by TRUS-guided saturation biopsy in two patients and by transurethral resection of the prostate due to bladder outlet obstruction in eight patients. Assuming these interval carcinomas were already present at the time of MRI intervention, the rate of missed cancers by MRI-guided prostate biopsy in our series was at most 10.4%.

Thirty-one of the patients with confirmed prostate cancer underwent radical prostatectomy in our institution. After review of the prostatectomy specimens by an experienced pathologist, we estimated the accuracy of MRI-guided core biopsies in detecting prostate cancer. In our patient cohort with histological confirmed prostate cancer, 138 biopsy cores were extracted, 72 from areas suspected of malignancy according to the above-mentioned MRI criteria, and 66 from areas that appeared normal. Malignancy was correctly observed in 69 biopsies, while three false-positive, 63 true-negative, and three false-negative results were observed (Table 2). These results yielded a sensitivity of 95.8% and a specificity of 95.5%, with a positive predictive value of 95.8% and a negative predictive value of 99.5% to 95.5%.

Next, we compared whether the location of areas suspected of malignancy matched those listed in the pathologist's final report. In the majority of cases, the location of prostate cancer was correctly determined by MR imaging. Only in 12 cases the pathologist's final report revealed a more widespread cancer than determined by MRI.

**Figure 2:**
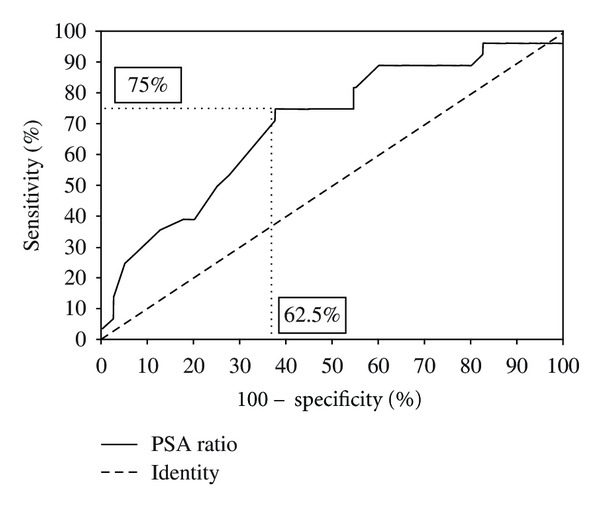
ROC curve for the cutoff value of the free-to-total PSA ratio, the specificity, and sensitivity for prostate cancer detection with the MRI-guided biopsy technique. The horizontal and vertical lines mark the sensitivity and specificity at the selected cutoff of 0.15.

The free-to-total PSA ratio was a strong predictor of prostate cancer detection by MRI-guided prostate biopsy. To determine the cutoff value of the free-to-total PSA ratio, the specificity and sensitivity for prostate cancer detection were calculated on the basis of the receiver operating characteristics (ROC) curve (area under the curve 0.70, 95% CI, 0.57–0.83, *P* = 0.0054; Figure 2). Applying this cutoff value, there was a highly significant difference in the cancer detection rate using MRI-guided prostate biopsy between males with a PSA ratio <0.15 compared to those with a PSA ratio ≥0.15 (*P* = 0.0073, Fisher's exact test). In 35 patients with a ratio <0.15, the prostate cancer detection rate was 57.1% (20 of 35), while in 33 patients with a ratio ≥0.15, prostate cancer was detected in only eight patients (24.2%; Table 3). Of the 10 patients with negative MRI-guided prostate biopsies, who revealed prostate cancer after MRI intervention, seven patients showed a free-to-total PSA ratio <0.15 (70%).

### 3.2. Clinical Complications

Twenty-three patients developed gross hematuria for less than six hours. Sixteen patients reported short-term perianal bleeding. Urinary retention requiring a single catheterization occurred in one patient. No patients were hospitalized for complications.

## 4. Discussion

Today, TRUS-guided prostate biopsy is the gold standard for the diagnosis of prostate cancer. When applied as a sextant biopsy in patients with a total PSA value ranging from 4–10 ng/mL, this approach yields a sensitivity of 39%–52% and a specificity of 81%-82% [11]. Yet, about 20% of prostate cancers are not detected at the first biopsy. If the first biopsy is negative, a repeat biopsy may be recommended, which has a cancer detection rate between 20% to 35% [12–15]. De la Taille has reported even higher cancer detection rates with extended biopsy schemes (more than 12 cores up to saturation biopsy schemes ≥20 cores) [16]. A meta-analysis studied the efficacy and adverse effects of various biopsy schemes and found that an extended biopsy with 12 cores strikes a balance between adequate cancer detection and an acceptable level of adverse effects. However, biopsies of more than 12 cores seemed to be of no significant benefit in cancer detection, and biopsies with 18 or more cores had a poor side-effect profile [17]. Targeted biopsies, directed by contrast enhanced doppler ultrasound, showed detection rates similar to those seen with systematic biopsies; nevertheless, this technique has not yet gained widespread acceptance not even in the repeat biopsy setting [18–20]. One study on repeat biopsies analyzed the value of saturation prostate biopsies, where 41 to 76 cores were taken per patient. In accordance with other studies, they reported a low diagnostic yield with a cancer detection rate of 11%, which is comparable to traditional biopsy schemes [21].

Due to dramatic improvements in MR imaging, this technology has gained growing importance in the diagnosis of prostate cancer. The capability of combining MR imaging with techniques to simultaneously perform a targeted biopsy of the prostate is of particular interest to urologists. The first report on MRI-guided biopsy of the prostate was published in 2000 describing a transperineal puncture in one patient in the lithotomy position under general anesthesia [4]. Soon, other reports described transrectal punctures with patients placed in a prone position [5, 7].

In our study cohort, consisting of 96 patients with one or more previous negative TRUS-guided biopsy sessions, prostate cancer was detected in 40.6% of the cases using MRI-guided targeted biopsy. This high rate of prostate cancer detection not only demonstrates the feasibility of MR imaging in combination with simultaneous prostate biopsy, but also the excellent performance of MR imaging in this difficult to treat patient cohort. For individual core biopsies, our technique yields a sensitivity of 95.8%, a specificity of 95.5%, a positive predictive value of 95.8%, and a negative predictive value of 95.5%. An even higher detection rate could be achieved when patients were stratified according to their free-to-total PSA ratio. The group with a PSA ratio <0.15 had a detection rate of 57.1% versus 24.2% for the group with a PSA ratio ≥0.15.

It is difficult to estimate the rate that the presence of prostate cancer was not detected. Yet, we determined a rough estimation from our follow-up evaluations of 57 men with negative MRI-guided biopsies. In 10 patients, prostate cancer was diagnosed within 3 years after the MRI intervention. Assuming these interval carcinomas were already present at the time of MRI intervention, the rate that cancer was missed is at most 10.4%. Following an initial negative saturation biopsy, a notably higher rate (24%) of subsequent prostate cancer detection during followup has been reported [22]. It is of particular note that in our series, seven of the 10 patients with an interval prostate cancer had a free-to-total PSA ratio <0.15 at the time of MRI intervention, further emphasizing the PSA ratio is an important predictor of prostate cancer.

The complications associated with MRI-guided prostate biopsy in our study, such as hematuria, perianal bleeding, or urinary retention, are comparable to those associated with TRUS-guided biopsies [23, 24], and their rates are similar to those reported in recent studies on MRI-guided biopsy techniques [6, 7, 25]. Major complications warranting hospitalization were not observed. This is in contrast to complications arising after saturation prostate biopsies, which include sepsis and occur at a rate of 12% [26].

Although MRI-guided prostate biopsy is time consuming, it can be recommended for patients with persisting prostate cancer suspicion despite a prior negative TRUS-guided biopsy. In our series of patients with prior negative prostate biopsies, we observed an improved prostate cancer detection rate of over 40% in MRI-guided prostate biopsy compared to 20% to 35% reported for conventional TRUS-guided repeat biopsies.

## Figures and Tables

**Table 1:** Patients' characteristics.

	Median (range)		Median		
Variable	All patients (*n* = 96)		Prostate cancer detected (*n* = 39)	No prostate cancer detected (*n* = 57)	*P* value^a^

Age (years)	66.2	(45.6–84)	67.3	65.9	0.69
Total serum PSA (ng/mL)	9.4	(1.0–48.8)	11.2	8.5	0.0039
Free serum PSA (ng/mL)^b^	1.2	(0.1–5.6)	1.3	1.1	0.78
Free/Total PSA ratio^b^	0.1	(0.04–0.5)	0.1	0.2	0.005
Prostate volume (mL)	37	(12–120)	34	40	0.081

^a^ *P* values of variables with or without prostate cancer detection were compared using Mann-Whitney *U*-test statistics.

^b^Free PSA measurements were available from 69 patients.

**Table 2:** Sensitivity and specificity.

	Pathological report	
MRI-guided bopisy	Tumor	Normal
Malignancy suspected	69	3
Malignancy not suspected	3	63

In a cohort of 31 patients with histological confirmed prostate cancer, a total of 138 biopsy cores were extracted.

**Table 3:** Relationship of free/total PSA ratio and prostate cancer detection by MRI-guided biopsy.

Free/total PSA ratio	Prostate cancer detected (%)
≥0.15 (*n* = 33)	8 (24.2)
<0.15 (*n* = 35)	20 (57.1)
